# Apelin and apelin receptor expression in renal cell carcinoma

**DOI:** 10.1038/s41416-019-0396-7

**Published:** 2019-02-20

**Authors:** Yuri Tolkach, Jörg Ellinger, Anika Kremer, Laura Esser, Stefan C. Müller, Carsten Stephan, Klaus Jung, Marieta Toma, Glen Kristiansen, Stefan Hauser

**Affiliations:** 1 Institute of Pathology, University Hospital Bonn, Sigmund-Freud-Strasse 25, 53105 Bonn, Germany; 2 Department of Urology, University Hospital Bonn, Sigmund-Freud-Strasse 25, 53105 Bonn, Germany; 3 Department of Urology, Charite - Universitätsmedizin Berlin, Schumannstrasse 20, 10117 Berlin, Germany; 4 Berlin Institute for Urologic Research, Schumannstrasse 20, 10117 Berlin, Germany

**Keywords:** Renal cell carcinoma, Predictive markers

## Abstract

**Background:**

The *APLNR* (apelin receptor) has been shown to be an essential gene for cancer immunotherapy, with deficiency in APLNR leading to immunotherapy failure. The aim of this study is to investigate the expression of APLN (apelin) and APLNR in patients with renal cell carcinoma (RCC), and its association with clinicopathological parameters and survival.

**Methods:**

Three well-characterised patient cohorts with RCC were used: Study cohort 1 (clear-cell RCC; *APLN/APLNR* mRNA expression; *n* = 166); TCGA validation cohort (clear-cell RCC; *APLN/APLNR* mRNA expression; *n* = 481); Study cohort 2 (all RCC subtypes; APLNR protein expression/immunohistochemistry; *n* = 300). Associations between mRNA/protein expression and clinicopathological variables/patients’ survival were tested statistically.

**Results:**

While APLN showed only very weak association with tumour histological grade (TCGA cohort), APLNR/mRNA protein expression correlate significantly with ccRCC aggressiveness. APLNR is expressed in tumour vasculature and tumour cells at different levels, and these expression levels associate with tumour aggressiveness in opposing directions. APLNR expression was negatively correlated with PD-L1 expression by tumour cells in a subset of patients with ccRCC. APLNR expression in either compartment is an independent prognostic factor for survival of patients with ccRCC.

**Conclusion:**

The APLNR/APLN-system appears to play an important role in ccRCC, warranting further clinical investigation.

## Background

Metastatic renal cell carcinoma (RCC) is a common oncological disease worldwide, that is resistant to radiation therapy and conventional cytotoxic chemotherapy.^1^ Immunotherapy has been a viable treatment since the 1990s, but the advent of immune checkpoint inhibition has improved therapy responses considerably.^2,3^ Predictive biomarkers for rational patient selection are currently under extensive investigation.^4^

Recently, the apelin receptor (coded by the *APLNR* gene) was identified as prerequisite for successful cancer immunotherapy, with APLNR deficiency associated with immunotherapy failure.^5^ Since apelin (APLN) and the apelin receptor have long been known to play important roles in vascular physiology,^6,7^ emerging evidence of their importance in oncological diseases is not surprising.^8–11^ Given the paucity of data concerning the role of APLN and APLNR in renal cell carcinoma^12^ and the importance of immune evasion mechanisms of this tumour, our study aimed to investigate the expression of APLN and APLNR in RCC and its association with clinicopathological parameters and survival.

## Materials and methods

### mRNA analysis (Study cohort 1)

With pre-operative written informed consent, renal tissues were collected from 166 patients within the framework of the tissue bank at the CIO Cologne-Bonn (clinical characteristics in Suppl. Table 1). Patients underwent radical or partial nephrectomy at the Department of Urology at the University Hospital Bonn.

Fresh-frozen tissues were stored at −80 °C prior to use. Total RNA was isolated from 166 ccRCC and 102 normal renal tissue samples as described before.^13^ In brief, total RNA was isolated with the mirVana miRNA Isolation Kit (Ambion, Foster City, CA, USA) and subsequently treated with DNase (DNA-free Kit, Ambion). The RNA quantity was determined using a NanoDrop 2000 spectrophotometer (Thermo Scientific, Wilmington, DE, USA). RNA integrity was confirmed by evaluation of the 28S and 18S rRNA bands in a gel electrophoresis.

The *APLN* and *APLNR* mRNA expression levels were determined using quantitative real-time PCR; *ACTB, GAPDH* and *PPIA* were used as reference genes (see Supplementary Table 2 for primer sequences). Relative expression levels were calculated using the 2^-ΔΔCT^ algorithm using the Applied Biosystems QuantStudio 3D Analysis Suite Cloud software. cDNA was synthesised from 1 µg total RNA using the PrimeScript RT Reagent Kit with gDNA Eraser (Takara Bio, Saint-Germain-en Laye, France). For each qPCR, 5 ng/µl cDNA was amplified with 1× SYBR Premix Ex Taq II and ROX Plus with 10 pmol/µl forward/reverse primer. PCR experiments were performed on the QuantStudio 5 Real-Time PCR System (Thermo Fisher Scientific).

### The Cancer Genome Atlas analysis (TCGA cohort)

Clinical data and normalised mRNA expression data generated with Illumina HiSeq 2000 RNA sequencing platform, version 2 (data version 28.01.2016) were extracted from TCGA for patients with clear-cell RCC. After database construction with thorough control of data quality, 481 patient cases with complete mRNA expression and clinical information were available for analysis.

### Immunohistochemistry analysis (Study cohort 2)

Three-hundred patients diagnosed with renal cell carcinoma after radical or partial nephrectomy at a single institution (Department of Urology, Charité—Universitätsmedizin Berlin, Germany; 1992–2004) were included in this study (Suppl. Table 1). The mean follow-up time was 117 months (total range: 1–267 months), allowing the calculation of overall survival as an endpoint. Formalin-fixed, paraffin-embedded archive tissue was used to construct a tissue microarray (TMA) with two tumours and two normal tissue spots (diameter 1 mm) from every patient. The tissue microarray was cut (3 µm thick) and mounted on superfrost slides (Menzel Gläser, Brunswick, Germany). After deparaffinisation with xylene and gradual rehydration, antigen retrieval was achieved by pressure-cooking in 0.01 mol/L citrate buffer for 5 min. Slides were incubated with primary antibody (APLNR rabbit polyclonal antibody, ThermoFisher Scientific, Catalogue number PA5-21285; Dilution 1:50). The slides were counterstained with haematoxylin and aqueously mounted. The immunohistochemical staining was evaluated blind to clinical outcome, clinical and pathological stage. Staining intensities were graded separately for cytoplasm and membrane of tumour cells and endothelial cells of tumour vessels. A 4-tier grading system (0: negative; 1: weakly positive; 2: moderately positive; 3: strongly positive) was used.

TMA Slides were also stained with CD34 antibody (monoclonal antibody, Dako/Agilent; Dilution 1:100, m7165) for microvessel density assessment and with PD-L1 antibody (monoclonal mouse anti-human antibody, Clone 22C3, Dako/Agilent; pharmDx kit). PD-L1 staining was evaluated by means of the percent of positive tumour cells in a TMA spot (membrane staining).

### Cell lines

The following cell lines were used for western blot: Caki-1 (human ccRCC), 786-0 (human ccRCC), RC-124 (human kidney adult primary cell line, benign), as well as DU145 and PC-3 prostate cancer cell lines. All cells were cultured in their specific media (786-0, DU-145 and PC-3: RPMI-1640; Caki-1: McCoy’s 5A medium; RC-124: DMEM GlutaMAX medium; all media Thermo Fisher Scientific). The media were supplemented with 10% foetal bovine serum (FBS) and 1% Penicillin/Streptomycin. The cells were grown in a humidified incubator at 37 °C with 5% CO_2_ and regularly tested for mycoplasma with negative outcome.

### Western blot/antibody validation

RIPA buffer (Cell Signalling) was used for cell lysis, according to the manufacturer’s instructions, and the protein concentrations were determined using a BCA Protein Assay Kit (Pierce). Proteins were separated by SDS-PAGE using Tris-Glycine 10% polyacrylamide gels in SDS page running buffer and transferred to methanol-activated PVDF membranes according to the standard protocol. Membranes were immunoblotted with antibodies against APLNR (Thermo Fisher, PA5-21285, 1:500) after blocking in 5% milk (nonfat dried milk powder, AppliChem Panreac, A0830). Following primary antibody incubation, membranes were probed with HRP-conjugated mouse anti-rabbit secondary antibody (Santa Cruz Biotechnology; sc-374015; Dilution 1:5000) and imaged using the Chemidoc system (BioRad). Lamin B1 (Santa Cruz Biotechnology, sc-374015) served as a loading control in a first experiment with Caki-1, 786-0, RC-124 and PC3 cell lines (Suppl. Figure 1). In the second experiment carried out using 786-0, PC3 and DU-145 in triplicates, ß-Actin (Santa Cruz, SC-47778, 1:200) was used as a loading control, probed with HRP-conjugated rabbit anti-mouse secondary antibody (Dako, P-0260, 1:5,000); for results see Fig. 3 and Suppl. Data 1.

### Microvessel density assessment

All TMA slices were digitalised at an objective magnification of 20× and saved in MIRAX-format. Automated microvessel density assessment was carried out using “Vessel detection” module in Tissue Studio of Definiens Developer XD software (v.2; Munich, Germany) on individual tumour spots (Suppl. Figure 2). Median microvessel densities (number of vessels/mm^2^) were calculated when more than one tumour spot was available for analysis.

### Ethical issues

The study was approved by the ethic committee at the University Bonn (vote: 317/17).

### Statistical analyses

All statistical analyses were made in R (version 3.2.3; The R Foundation for Statistical Computing). The follow-up period for overall survival analyses in the immunohistochemistry cohort (study cohort 2) was limited to 180 months. Optimal cut-off values for mRNA expression data were determined using the *survMisc* package for R (based on consecutive evaluation of all available cut-offs using univariate Cox regression).

## Results

### mRNA expression analyses (mRNA cohort)

**Fig. 1:**
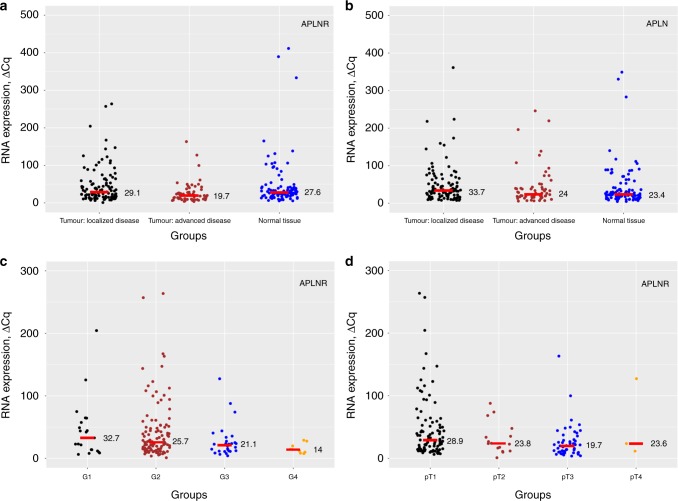
**a** *APLNR* mRNA expression in normal renal tissue and in clear-cell renal cell carcinoma (ccRCC) tumour tissue (patients with localised and advanced disease). **b** *APLN* mRNA expression in normal renal tissue and in ccRCC tumour tissue (patients with localised and advanced disease). **c** Association between *APLNR* mRNA expression and histological grade of the tumour, ccRCC: lower expression in higher grade tumours (*p* = 0.027). **d** Association between *APLNR* mRNA expression and pT-stage of the tumour, ccRCC: lower expression in higher stage tumours (*p* = 0.009). For all plots: horizontal red line and associated value implies median of the expression in group

*APLN* (*p* = 0.110) and *APLNR* (*p* = 0.105) mRNA expression levels were similar in normal (*n* = 102) and malignant tissues (ccRCC, *n* = 166) (Fig. 1). *APLNR* expression was inversely correlated with histological grade of the tumour (Pearson’s *r* = −0.17, *p* = 0.027), pT-stage (Pearson’s *r* = −0.20, *p* = 0.009) and presence of metastatic disease (Pearson’s *r* = -0.20, *p* = 0.009), while *APLN* mRNA expression showed no significant correlation.

**Fig. 2:**
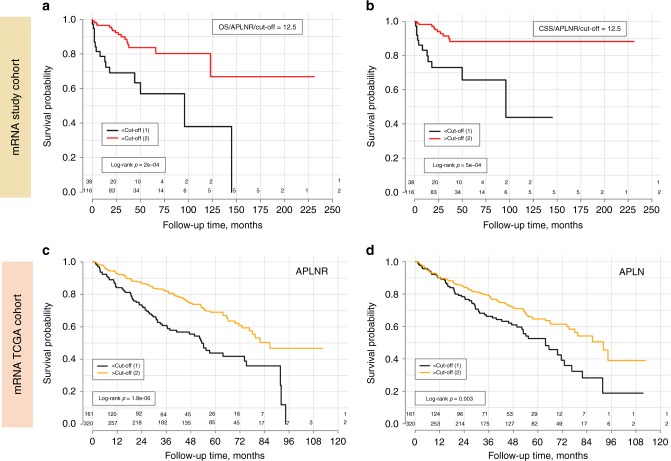
Survival analysis (Kaplan–Meier estimates) for *APLNR* and *APLN* mRNA expression in patients with clear-cell renal cell carcinoma. **a** mRNA study cohort: *APLNR* mRNA expression dichotomised using optimised expression cut-off (∆Cq = 12.5), overall survival as endpoint. **b** mRNA study cohort: *APLNR* mRNA expression dichotomised using optimised expression cut-off (∆Cq = 12.5), cancer-specific survival as endpoint. **c** mRNA TCGA cohort: *APLNR* mRNA expression dichotomised using optimised expression cut-off (number of reads RNAseq = 853), overall survival as endpoint. Also, statistically significant when median used as cut-off (*p* = 0.004). **d** mRNA TCGA cohort: *APLN* mRNA expression dichotomised using optimised expression cut-off (number of reads RNAseq = 1561), overall survival as endpoint. Not statistically significant when median used as cut-off (*p* = 0.113)

In the survival analysis (*n* = 154, number of events: OS = 31, CSS = 21) *APLN* expression showed no prognostic association with either overall survival or cancer-specific survival. In contrast, *APLNR* was predictive for overall (OS) and cancer-specific survival (CSS) in Kaplan–Meier (Fig. 2a, b), univariate (OS: HR 3.5, 95% CI 1.7–7.0, *p* = 0.0006; CSS: HR 4.1, 95% CI 1.7–9.7, *p* = 0.001) and multivariate Cox analyses of mRNA expression, histological grade and pT-stage (OS: HR 2.9, 95% CI 1.4–5.8, *p* = 0.004; CSS: HR 3.5, 95% CI 1.5–8.4, *p* = 0.001), with lower expression predicting shorter survival times.

### mRNA expression analyses (TCGA cohort)

In the TCGA ccRCC cohort, *APLNR* mRNA expression levels were negatively correlated with histological grade (Pearson’s *r* = −0.22, *p* = 5.2e−07) and pT-stage of the tumour (Pearson’s *r* = −0.23, *p* = 2.7e−07). *APLN* mRNA expression was only weakly inversely correlated with histological grade (Pearson’s *r* *=* −0.10, *p* = 0.036) and positively with APLNR mRNA expression (Pearson’s *r* *=* 0.10, *p* = 0.022).

Both low *APLNR* and low *APLN* mRNA expression levels were predictive of lower overall survival in patients with ccRCC in Kaplan–Meier (Fig. 2c, d) and in univariate Cox analysis with dichotomisation based on the optimised cut-off values (*APLNR*: HR 2.1, 95% CI 1.6–2.9, *p* = 3.1e−06; *APLN*: HR 1.6, 95% CI 1.2–2.2, *p* = 0.004). However, independent prognostic significance in multivariate Cox analysis (gene mRNA expression, histologic grade, pT-stage, pN-status) was only observed for APLNR expression (HR 1.8, 95% CI 1.3–2.5, *p* = 0.0009; see also Suppl. Table 3).

### Antibody validation

**Fig. 3:**
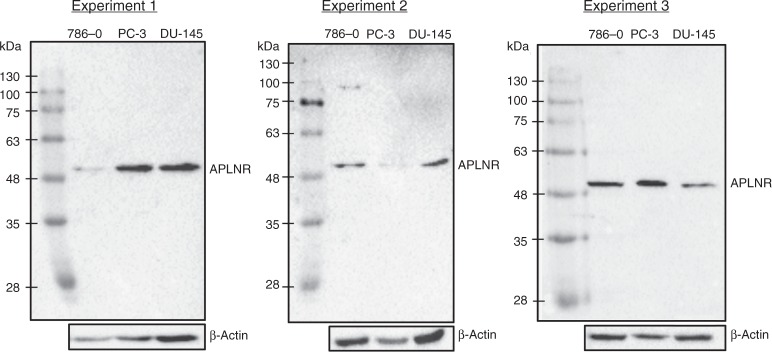
Western blotting results with the use of apelin receptor (APLNR) as validation target and β-actin antibody as load control (triplicate measurements under the same conditions). Following cell lines were used for western blot: 786-0 (human ccRCC), PC-3 and DU-145 (both prostate cancer cell lines)

Results of western blot experiments are outlined in Fig. 3 (associated metrics in Supplementary Data 1) and Suppl. Figure 1. A band with a molecular weight similar to that predicted for APLNR (~43 kDa) ±10% was detected in all cell lines used.

### Immunohistochemistry analysis (Immunohistochemistry cohort)

#### Staining patterns

**Fig. 4:**
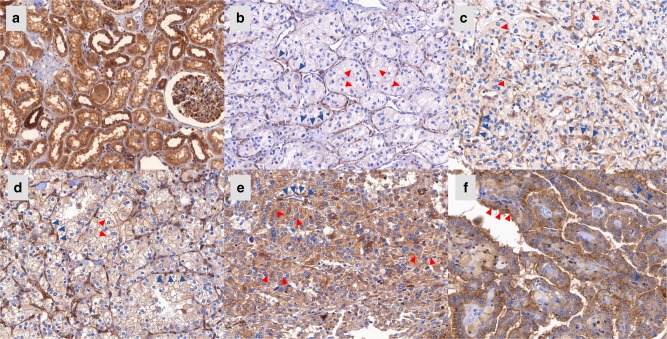
Staining patterns in normal and tumour tissue, Apelin receptor (APLNR). All microphotographs have magnification 200×. **a** Normal renal tissue: strong epithelial staining. **b** clear-cell renal cell carcinoma (ccRCC), Tumour cells: cytoplasm negative (“0”), Tumour vasculature: weak to moderate staining intensity (“1”-“2”). **c** ccRCC, Tumour cells: cytoplasm weak staining intensity (“1”), Tumour vasculature: moderate staining intensity (“2”). **d** ccRCC, Tumour cells: cytoplasm moderate staining intensity (“2”), also cell membrane moderate intensity; Tumour vasculature: strong staining intensity (“3”). **e** ccRCC, Tumour cells: cytoplasm strong staining (“3”), Tumour vasculature: moderate staining (“2”). **f** papillary RCC, Tumour cells: cytoplasm moderate staining intensity (“2”), also cell membrane strongly positive (“3”); Tumour vasculature: single endothelial cells positive. Red arrowheads point at epithelial cells, dark blue arrowheads point at tumour vessels

Normal tissue demonstrated almost uniformly very high expression of the APLNR protein (Fig. 4). In tumour tissue, expression of APLNR was detectable in tumour cells (cytoplasm and membrane, highly correlated; Pearson’s *r* = 0.46, *p* = 8.8e−15) and on endothelia of tumour vessels (no significant correlation with staining of tumour cells), see Fig. 4. While cytoplasmic staining of tumour cells was negatively associated with overall patient survival, vascular staining associated positively with overall patient survival.

#### Association with clinicopathological variables

Papillary RCC (pRCC) and chromophobe RCC (chRCC) tissue samples demonstrated relatively low vascular and relatively high cytoplasmic APLNR expression in tumour cells, while clear-cell RCC (ccRCC) showed a wide spectrum of staining intensities in both cytoplasm of tumour cells and endothelium (Suppl. Table 4). Because of the small number of patients with pRCC or chRCC, we restricted further analyses to ccRCC (*n* = 253).

Endothelial expression in tumour vessels was significantly and inversely associated with histological grade of the tumour and pT-stage (Table 1), and not associated (*p* > 0.05) with pN-status, gender, age or status at the end of the follow-up (overall survival).

**Table 1:** Immunohistochemistry cohort: associations between APLNR protein expression and clinicopathological parameters in clear-cell RCC (*n* = 253)

	Tumour vascular expression, staining intensity				Tumour cell cytoplasm expression, staining intensity			
	“0”	“1”	“2”	“3”	“0”	“1”	“2”	“3”
*Histological grade (WHO 2016), n (%)*								
G1	1 (2.0%)	17 (33.3%)	17 (33.3%)	16 (31.4%)	7 (13.7%)	34 (66.7%)	8 (15.7%)	2 (3.9%)
G2	5 (3.5%)	45 (31.7%)	56 (39.4%)	36 (25.3%)	15 (10.6%)	73 (51.4%)	38 (26.8%)	16 (11.3%)
G3	3 (7.6%)	14 (35.8%)	14 (35.8%)	8 (20.5%)	1 (2.6%)	11 (28.2%)	17 (43.6%)	10 (25.6%)
G4	9 (42.9%)	9 (42.9%)	1 (4.7%)	2 (9.5%)	3 (14.3%)	8 (38.1%)	7 (33.3%)	3 (14.3%)
p-level^a^			3.6e−08				0.002	
*pT-stage, n (%)*								
pT1	4 (2.7%)	52 (34.9%)	48 (32.2%)	45 (30.2%)	14 (9.4%)	81 (54.4%)	41 (27.5%)	13 (8.7%)
pT2	3 (17.6%)	2 (11.8%)	10 (58.8%)	2 (11.8%)	0 (0%)	7 (41.2%)	8 (47.1%)	2 (11.8%)
pT3 + pT4	11 (12.6%)	31 (35.6%)	30 (34.5%)	15 (17.2%)	12 (13.8%)	38 (43.7%)	21 (24.1%)	16 (18.4%)
p-level^a^			0.002				0.08	

^a^Pearson’s Chi-squared test

Cytoplasmic tumour cell expression was significantly and positively associated with tumour grade but was not with pT-stage, pN-status, gender or age (all *p* > 0.05).

#### Microvessel density

Median microvessel density (microvessels/mm^2^) for patients with ccRCC was 740, with pRCC 402 and with chRCC 263. Microvessel density was negatively correlated with ISUP grade of the tumour (Pearson’s *r* *=* 0.22, *p* = 0.0003) and was positively correlated with intensity of vascular APLNR expression (Pearson’s *r* *=* 0.21, *p* = 0.0002).

#### Correlation with PD-L1 expression

PD-L1 immunohistochemistry protein expression status was available for 72 patients with ccRCC with a range of 0–100% tumour cells positive for PD-L1 (median 15%). Of these patients 23 (31.9%) were completely negative, 15 other patients showed low levels of expression (<10%) in at least 1 tumour spot. A negative correlation was evident between APLNR expression and PD-L1 expression by tumour cells: for cytoplasmic APLNR expression: Pearson’s *r* = −0.16, *p* < 0.001, for vascular expression: Pearson’s *r* = −0.19, *p* < 0.001.

#### Association with survival (ccRCC)

**Fig. 5:**
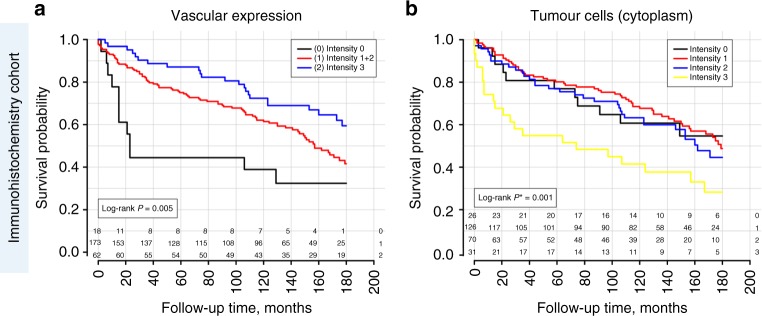
Survival analysis (Kaplan–Meier estimates) for APLNR protein expression in patients with clear-cell renal cell carcinoma (immunohistochemistry cohort), overall survival as endpoint. **a** APLNR expression on tumoural vessels; staining intensity: “0”—negative, “1”—weak expression, “2”—moderate expression, “3”—strong expression. **b** APLNR expression in cytoplasm of tumour cells; staining intensity (“0” negative, “1” weak expression, “2” moderate expression, “3” strong expression)

Endothelial expression in tumour vessels was significantly associated with overall survival in Kaplan–Meier/log-rank (Fig. 5a) und univariate Cox-analysis (not shown), but completely lost its significance in multivariate Cox-analysis due to interaction with histological grade and pT-stage of the tumour.

Cytoplasmic tumour cell expression was significantly associated with overall survival in Kaplan–Meier analysis (Fig. 5b), univariate (Intensity “3” vs. Intensity “0–2” HR 2.12, 95% CI 1.33–3.40, *p* = 0.002) and multivariate (HR 1.68, 95% CI 1.02–2.78, *p* = 0.041) Cox-analysis of histological grade, pT-stage, R-status, and ECOG performance status to account for other mortality causes (Suppl. Table 5).

## Discussion

The apelin receptor is a G-protein-coupled receptor which binds a number of substances (apelin, APELA, ELABELA, Toddler) with many important functions in the cardio-vascular system, such as cardiac development, vasomotor tone, angiogenesis, myocardial inotropy, prevention of fibrosis and remodelling.^6,7,14–17^ Recently, it was identified as an essential gene for cancer immunotherapy, which can modulate interferon-γ responses in tumours and the effector function of CD8^+^T cells.^5^ The loss of its function could reduce the efficacy of cancer immunotherapies.^5^

To our knowledge, only one screening study has addressed the expression of *APLN* (but not *APLNR*) in ccRCC tumour tissue and adjacent normal tissue, with the only finding that APLN mRNA expression was higher in tumour tissue.^12^ In our study we have used three well-characterised cohorts of patients to perform a comprehensive evaluation of mRNA and protein expression of *APLN* and *APLNR* in primary RCC tumours and to correlate tumour characteristics and patient survival.

At the mRNA level, we have demonstrated that *APLNR* expression is decreased in higher grade, higher stage and metastatic ccRCC tumours with independent prognostic significance for overall and cancer-specific survival. Immunohistochemical analysis of APLNR protein expression has provided important information concerning which compartments of the tumour tissue express the receptor. Both endothelial lining of the tumoural vessels and tumour cells express significant amounts of APLNR with relatively high variability between the cases. In support of our mRNA findings, we observe by immunohistology that APLNR expression in the vasculature and tumour cells associates inversely with tumour aggressiveness (pT-stage and grade as surrogates). Increasing aggressiveness is accompanied with higher APLNR expression in tumour cells and lower expression in vessels. Both expression parameters were associated with overall survival of patients, but only cytoplasmic expression in tumour cells associated independently in a multivariate model with common clinicopathological variables. Interestingly, vascular APLNR expression was positively associated with microvessel density, which itself correlates negatively to tumour aggressiveness (ISUP grade). The western blots from ccRCC cell lines support this finding (Fig. 3).

*APLN* mRNA expression showed weak association to histological grade of the tumour only in the TCGA cohort, in contrast the influence on overall survival showed strong association but failed as an independent predictor.

Little is known about the role of APLNR/APLN axis during oncogenesis/tumour growth. In colon adenocarcinomas, it has been shown to be upregulated together with its ligand APLN, forming a putative autocrine loop, stimulating tumour growth.^10^ Apelin has been shown to promote lymphangiogenesis and lymph node metastasis in experimental models with melanoma cells.^11^ In a cholangiocarcinoma cell model and in in vitro/in vivo experiments with glioblastoma cells, inhibition of the APLN/APLNR axis has resulted in decreased proliferation and angiogenesis.^8,18^ The role of the APLNR-axis for tumour neo-angiogenesis, especially under hypoxia, regulated by hypoxia-inducible factor (HIF)-1alpha, is well documented.^9,19,20^ ccRCC is a highly vascularised tumour with high levels of intratumoural HIF, which accumulates due to inactivation of the von Hippel-Lindau gene.^21^ This could explain the generally high vascular expression of the APLNR and APLN in tumour tissues in our study. However, it is unclear why increasing aggressiveness of the tumour leads to decreased levels of APLNR expression. One possible explanation could be that APLN/APLNR activation induces maturation of the tumour vasculature and improves the efficiency of immune therapy, while immature vessels could help the tumour to evade the immune response.^22^ Also, studies in glioblastoma suggest that release of APLN through endothelial cells triggers the response reactions from tumour cells expressing APLNR, so that an immuno-protective environment is created.^18^ However, the immunological effects of the APLN/APLNR axis in ccRCC and other tumours are only incompletely understood and probably involve a complex interplay between vascular and cellular compartments of the tumour. Our analysis of microvessel density, which is associated both with aggressiveness of the tumour (ISUP grade) and vascular expression of APLNR, once more supports this point. Importantly, while there is only one known isoform of APLNR, several different isoforms of APLN arise upon cleavage by endopeptidases and show different activity levels as well as different degrees of organ-specificity.^17^ These forms should be considered in further analyses.

Our study is limited. We have only used primary tumours for analysis. It would be interesting to compare the expression of APLNR/APLN in paired samples from primary tumours and metastases. Although our study provides the first thorough characterisation of APLN/APLNR expression in renal cell carcinoma (especially in ccRCC as the dominant subtype) and its association with clinicopathological variables and outcome, functional studies, especially those related to the associated immune processes, were not within the scope of our project and warrant further investigations.

Interestingly, in our study we were able to detect significant levels of negative correlation between APLNR expression in different tumour tissue compartments and PD-L1 expression by tumour cells in a subset of patients with ccRCC. This finding once more time underlines the potential relevance of APLNR for intratumoural immunological processes. The evaluation of treatment response to immune therapies (checkpoint inhibitors) as a function of APLNR expression should be investigated in future studies.

## Conclusions

In our study, we provide a comprehensive characterisation of APLNR and APLN expression in renal cell carcinoma. The main results are: (1) the significant correlation between ccRCC aggressiveness and APLNR mRNA/protein expression, (2) characterisation of different APLNR-expressing compartments in tumour tissue (tumoural vessels and tumour cells) with opposite correlations to tumour aggressiveness, (3) evidence of independent prognostic role of APLNR expression regarding patient survival, (4) correlation with PD-L1 expression by tumour cells in ccRCC.

## Supplementary information

Supplementary Data 1

Suppl. Table 1

Suppl. Table 2

Suppl. Table 3

Suppl. Table 4

Suppl. Table 5

Suppl Figure 1

Suppl Figure 2

Database mRNA

Database IHC

## Data Availability

Study databases for mRNA cohort and immunohistochemistry cohort are available in electronic form as Supplementary data (Database IHC.xls, Database mRNA.xls). TCGA datasets used for in silico analyses were downloaded from the Broad Institute Firehose GDAC archive (https://gdac.broadinstitute.org/).
