# Supplementary material for: Apelin and apelin receptor expression in renal cell carcinoma
Source: Br J Cancer. 2019 Feb 20;120(6):633–9. doi: 10.1038/s41416-019-0396-7 (PMC6461937; doi:10.1038/s41416-019-0396-7)
Supplement: Supplementary file 1 — Supplementary Data 1 [file 41416_2019_396_MOESM1_ESM.docx]

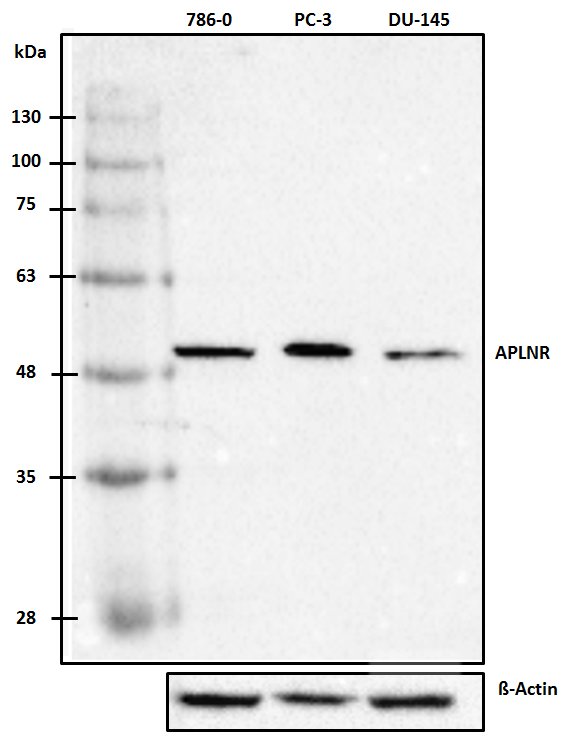


**WB 3**


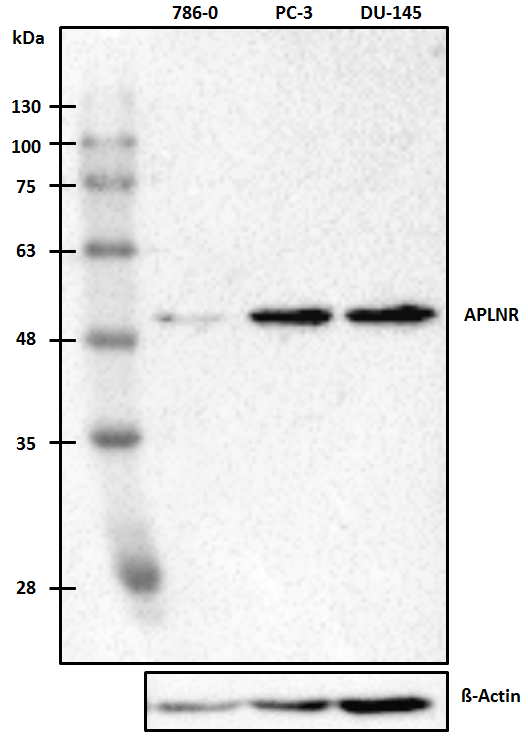


**WB 1**


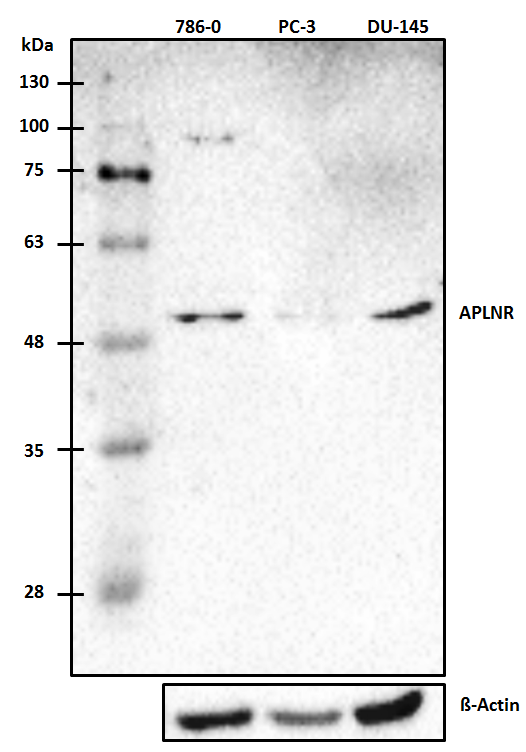


**WB 2**

| **WB1 APLNR** | | | | | | |  | **WB1 ß-Actin** | | | | | | |  |  |
| --- | --- | --- | --- | --- | --- | --- | --- | --- | --- | --- | --- | --- | --- | --- | --- | --- |
|  | Band | Mol. Wt. (KDa) | Relative Front | Rel. Quant. | Band % | Lane % |  |  | Band | Mol. Wt. (KDa) | Relative Front | Rel. Quant. | Band % | Lane % |  | Normalized Quant. |
| BlueStar prestained protein marker | 1 | 130.00 | 0.05 | 0.21 | 1.31 | 0.73 |  | BlueStar prestained protein marker | 1 | 130.00 | 0.06 | 0.06 | 1.49 | 1.31 |  |  |
|  | 2 | 100.00 | 0.11 | 0.81 | 5.09 | 2.82 |  |  | 2 | 100.00 | 0.14 | 0.25 | 5.83 | 5.12 |  |  |
|  | 3 | 75.00 | 0.17 | 1.44 | 9.08 | 5.03 |  |  | 3 | 75.00 | 0.20 | 0.24 | 5.52 | 4.85 |  |  |
|  | 4 | 63.00 | 0.26 | 2.17 | 13.64 | 7.55 |  |  | 4 | 63.00 | 0.32 | 0.62 | 14.44 | 12.68 |  |  |
|  | 5 | 48.00 | 0.39 | 2.36 | 14.82 | 8.20 |  |  | 5 | 48.00 | 0.46 | 1.05 | 24.73 | 21.71 |  |  |
|  | 6 | 35.00 | 0.53 | 3.20 | 20.15 | 11.15 |  |  | 6 | 35.00 | 0.62 | 0.97 | 22.81 | 20.03 |  |  |
|  | 7 | 28.00 | 0.73 | 5.71 | 35.92 | 19.88 |  |  | 7 | 28.00 | 0.84 | 1.07 | 25.18 | 22.10 |  |  |
| 786-0 | 1 | 51.06 | 0.36 | 1.00 | 100.00 | 6.37 |  | 786-0 | 1 | 44.64 | 0.46 | 1.00 | 100.00 | 57.99 |  | 1.00 |
| PC-3 | 1 | 51.70 | 0.36 | 7.46 | 100.00 | 14.72 |  | PC-3 | 1 | 45.07 | 0.46 | 1.96 | 100.00 | 75.83 |  | 3.81 |
| DU-145 | 1 | 51.70 | 0.36 | 8.72 | 100.00 | 31.01 |  | DU-145 | 1 | 45.07 | 0.46 | 3.60 | 100.00 | 90.29 |  | 2.42 |
|  |  |  |  |  |  |  |  |  |  |  |  |  |  |  |  |  |
| **WB2 APLNR** | | | | | | |  | **WB2 ß-Actin** | | | | | | |  |  |
|  | Band | Mol. Wt. (KDa) | Relative Front | Rel. Quant. | Band % | Lane % |  |  | Band | Mol. Wt. (KDa) | Relative Front | Rel. Quant. | Band % | Lane % |  |  |
| BlueStar prestained protein marker | 1 | 130.00 | 0.08 | 0.64 | 10.62 | 10.00 |  | BlueStar prestained protein marker | 1 | 130.00 | 0.02 | 0.01 | 0,452736 | 0,423174 |  |  |
|  | 2 | 100.00 | 0.17 | 0.12 | 1.93 | 1.82 |  |  | 2 | 100.00 | 0.18 | 0.05 | 2.68 | 2.51 |  |  |
|  | 3 | 75.00 | 0.24 | 1.30 | 21.50 | 20.24 |  |  | 3 | 75.00 | 0.26 | 0.13 | 6.67 | 6.23 |  |  |
|  | 4 | 63.00 | 0.35 | 0.60 | 9.93 | 9.35 |  |  | 4 | 63.00 | 0.36 | 0.27 | 13.21 | 12.34 |  |  |
|  | 5 | 48.00 | 0.50 | 0.70 | 11.64 | 10.96 |  |  | 5 | 48.00 | 0.51 | 0.35 | 17.35 | 16.22 |  |  |
|  | 6 | 35.00 | 0.66 | 0.85 | 14.12 | 13.29 |  |  | 6 | 35.00 | 0.68 | 0.44 | 21.65 | 20.23 |  |  |
|  | 7 | 28.00 | 0.88 | 1.82 | 30.27 | 28.50 |  |  | 7 | 28.00 | 0.89 | 0.76 | 37.99 | 35.51 |  |  |
| 786-0 | 1 | 51.53 | 0.46 | 1.00 | 100.00 | 27.62 |  | 786-0 | 1 | 48.49 | 0.51 | 1.00 | 100.00 | 80.33 |  | 1.00 |
| PC-3 | 1 | 51.53 | 0.46 | 0.40 | 100.00 | 8.66 |  | PC-3 | 1 | 48.49 | 0.51 | 0.75 | 100.00 | 72.68 |  | 0.53 |
| DU-145 | 1 | 52.14 | 0.45 | 1.11 | 100.00 | 33.25 |  | DU-145 | 1 | 49.47 | 0.50 | 1.26 | 100.00 | 82.31 |  | 0.88 |
|  |  |  |  |  |  |  |  |  |  |  |  |  |  |  |  |  |
| **WB3 APLNR** | | | | | | |  | **WB3 ß-Actin** | | | | | | |  |  |
|  | Band | Mol. Wt. (KDa) | Relative Front | Rel. Quant. | Band % | Lane % |  |  | Band | Mol. Wt. (KDa) | Relative Front | Rel. Quant. | Band % | Lane % |  |  |
| BlueStar prestained protein marker | 1 | 130.00 | 0.18 | 0.18 | 5.60 | 5.03 |  | BlueStar prestained protein marker | 1 | 130.00 | 0.14 | 0.05 | 2.80 | 2.52 |  |  |
|  | 2 | 100.00 | 0.25 | 0.23 | 7.12 | 6.40 |  |  | 2 | 100.00 | 0.21 | 0.13 | 6.97 | 6.29 |  |  |
|  | 3 | 75.00 | 0.32 | 0.19 | 6.11 | 5.49 |  |  | 3 | 75.00 | 0.28 | 0.09 | 4.70 | 4.24 |  |  |
|  | 4 | 63.00 | 0.42 | 0.46 | 14.49 | 13.02 |  |  | 4 | 63.00 | 0.39 | 0.29 | 15.18 | 13.71 |  |  |
|  | 5 | 48.00 | 0.56 | 0.47 | 14.87 | 13.36 |  |  | 5 | 48.00 | 0.54 | 0.34 | 17.45 | 15.76 |  |  |
|  | 6 | 35.00 | 0.71 | 0.75 | 23.63 | 21.23 |  |  | 6 | 35.00 | 0.70 | 0.45 | 23.36 | 21.09 |  |  |
|  | 7 | 28.00 | 0.92 | 0.89 | 28.19 | 25.32 |  |  | 7 | 28.00 | 0.92 | 0.57 | 29.54 | 26.67 |  |  |
| 786-0 | 1 | 50.92 | 0.53 | 1.00 | 100.00 | 59.32 |  | 786-0 | 1 | 47.48 | 0.54 | 1.00 | 100.00 | 71.65 |  | 1.00 |
| PC-3 | 1 | 51.53 | 0.52 | 1.02 | 100.00 | 79.13 |  | PC-3 | 1 | 48.00 | 0.54 | 0.68 | 100.00 | 78.57 |  | 1.51 |
| DU-145 | 1 | 50.92 | 0.53 | 0.51 | 100.00 | 63.34 |  | DU-145 | 1 | 47.48 | 0.54 | 1.00 | 100.00 | 83.04 |  | 0.51 |

**WB 1**
