# Supplementary material for: Apelin and apelin receptor expression in renal cell carcinoma
Source: Br J Cancer. 2019 Feb 20;120(6):633–9. doi: 10.1038/s41416-019-0396-7 (PMC6461937; doi:10.1038/s41416-019-0396-7)
Supplement: Supplementary file 2 — Suppl. Table 1 [file 41416_2019_396_MOESM2_ESM.docx]

**Supplementary Table 1:**

Clinicopathological parameters of the study cohorts

|  | **mRNA cohort** | | **Immunohistochemistry cohort, n=300** | |
| --- | --- | --- | --- | --- |
|  | **ccRCC** | **normal** |  |  |
|  | **n=166 (%)** | **n=102 (%)** | **n** | **%** |
| **Sex** |  |  |  |  |
| male | 108 (65.1) | 71 (69.6) | 201 | 67% |
| female | 58 (34.9) | 31 (30.4) | 99 | 33% |
| **Age, years** |  |  |  |  |
| mean | 64.4 | 63.6 | 60.8 | - |
| min-max | 27-89 | 36-89 | 30-86 | - |
| **Pathological stage** |  |  |  |  |
| pT1 | 95 (57.2) | n.a. | 175 | 58.3% |
| pT2 | 16 (9.6) | n.a. | 25 | 8.3% |
| pT3 | 52 (31.3) | n.a. | 98 | 32.7% |
| pT4 | 3 (1.8) | n.a. | 2 | 0.7% |
| LN metastasis | 5 (3.0) | n.a. | 19 | 6.3% |
| distant metastasis | 22 (13.2) | n.a. | n.a. | - |
| **Grading** |  |  |  |  |
| grade 1 | 21 (12.6) | n.a. | 54 | 18.8% |
| grade 2 | 108 (65.1) | n.a. | 161 | 55.9% |
| grade 3 | 28 (16.9) | n.a. | 52 | 18.1% |
| grade 4 | 9 (5.4) | n.a. | 21 | 7.3% |
| **ECOG peformance status** |  |  |  |  |
| 0 | - | n.a. | 202 | 67.3% |
| 1 | - | n.a. | 88 | 29.3% |
| 2 | - | n.a. | 10 | 3.3% |
| **Histological subtype** |  |  |  |  |
| ccRCC | 166 (100%) | n.a. | 253 | 84.3% |
| pRCC | 0 (0%) | n.a. | 35 | 11.7% |
| chrRCC | 0 (0%) | n.a. | 12 | 4.0% |
| **R-status** |  |  |  |  |
| R0/Rx | 151 (91.0%) | n.a. | 283 | 94.3% |
| R1 | 15 (9.0%) | n.a. | 17 | 5.7% |

Comments: LN, Lymph node; n.a., not applicable; ccRCC, clear-cell renal cell carcinoma; pRCC, papillary RCC; chrRCC, chromophobe RCC.
