# Supplementary material for: Apelin and apelin receptor expression in renal cell carcinoma
Source: Br J Cancer. 2019 Feb 20;120(6):633–9. doi: 10.1038/s41416-019-0396-7 (PMC6461937; doi:10.1038/s41416-019-0396-7)
Supplement: Supplementary file 3 — Suppl. Table 2 [file 41416_2019_396_MOESM3_ESM.docx]

**Supplementary Table 2:**

Primer sequences for PCR-experiments

| **Gene** | **Forward Primer** | **Reverse Primer** |
| --- | --- | --- |
| ACTB | CCA ACC GCG AGA AGA TGA | CCA GAG GCG TAC AGG GAT AG |
| GAPDH | CTC TGC TCC TCC TGT TCG AC | ACG ACC AAA TCG GTT GAC TC |
| PPIA | ATG CTG GAC CCA AAC ACA AAT | TCT TTC ACT TTG CCA AAC ACC |
| APLN | CTG TCA GTT GGA CCC CAC AC | CCA ATG TGC CCT GTC TGG AC |
| APLNR | TTC TCT CCA CTC CCC AGC AT | CCC GAG GAT TTC CAG TCT GT |
