# Supplementary material for: Apelin and apelin receptor expression in renal cell carcinoma
Source: Br J Cancer. 2019 Feb 20;120(6):633–9. doi: 10.1038/s41416-019-0396-7 (PMC6461937; doi:10.1038/s41416-019-0396-7)
Supplement: Supplementary file 4 — Suppl. Table 3 [file 41416_2019_396_MOESM4_ESM.docx]

**Supplementary Table 3**:

TCGA cohort: Multivariate Cox analysis for dichotomized APLNR mRNA expression in patients with ccRCC (Endpoint: overall survival, n = 473, number of events = 157)

|  | **Hazard ratio (HR)** | **95% CI** | **p-level** |
| --- | --- | --- | --- |
| **APLNR mRNA**  **expression*** |  |  |  |
| High (>cut-off) | 1.0 | - | - |
| Low (>cut-off) | 1.8 | 1.3-2.5 | 0.0009 |
| **Histological grade (WHO 2016)**** |  |  |  |
| G2 | 1.0 | - | - |
| G3 | 1.3 | 0.8-2.0 | 0.181 |
| G4 | 2.7 | 1.7-4.5 | 6.2e-05 |
| **pT-stage** |  |  |  |
| pT1 | 1.0 | - | - |
| pT2 | 1.0 | 0.6-1.8 | 0.991 |
| pT3 | 2.4 | 1.6-3.6 | 1.6e-05 |
| pT4 | 4.5 | 1.8-11.0 | 0.001 |
| **pN-stage** |  |  |  |
| pN0 / cN0 | 1.0 | - | - |
| pN+ | 0.9 | 0.5-2.5 | 0.832 |

Comments: ccRCC, clear-cell renal cell carcinoma (RCC).* - Dichotomisation is based on the best cut-off for mRNA expression determined by RNAseq (cut-off number of reads = 1561). ** G1 patients (n=8) were excluded from this analysis as they uniformly confined the excellent survival and herewith distorted the statistics for G2-G4.
