# Supplementary material for: Apelin and apelin receptor expression in renal cell carcinoma
Source: Br J Cancer. 2019 Feb 20;120(6):633–9. doi: 10.1038/s41416-019-0396-7 (PMC6461937; doi:10.1038/s41416-019-0396-7)
Supplement: Supplementary file 5 — Suppl. Table 4 [file 41416_2019_396_MOESM5_ESM.docx]

**Supplementary Table 4**:

Immunohistochemistry cohort: APLNR protein expression in different histological RCC subtypes

|  | Maximal cytoplasmic expression, staining intensity | | | | Minimal vascular expression,  staining intensity | | | |
| --- | --- | --- | --- | --- | --- | --- | --- | --- |
|  | **0** | **1** | **2** | **3** | **0** | **1** | **2** | **3** |
| **ccRCC,**  **n (%)** | 26  (10.3%) | 126  (49.8%) | 70  (27.7%) | 31  (12.3%) | 18  (7.1%) | 85  (33.6%) | 88  (34.8%) | 62  (24.5%) |
| **pRCC,**  **n (%)** | 1  (2.8%) | 9  (25.7%) | 13  (37.1%) | 12  (34.3%) | 8  (22.9%) | 23  (65.7%) | 3  (8.6%) | 1  (2.9%) |
| **chrRCC,**  **n (%)** | 1  (8.3%) | 0  (0%) | 7  (58.3%) | 4  (33.3%) | 0  (0%) | 11  (91.7%) | 0  (0%) | 1  (8.3%) |
| *p-level** | 2.2e-05 | | | | 1.3e-08 | | | |

Comments: ccRCC, clear-cell renal cell carcinoma (RCC); pRCC, papillary RCC; chrRCC, chromophobe RCC; * - Fisher’s exact test.
