# Supplementary material for: Apelin and apelin receptor expression in renal cell carcinoma
Source: Br J Cancer. 2019 Feb 20;120(6):633–9. doi: 10.1038/s41416-019-0396-7 (PMC6461937; doi:10.1038/s41416-019-0396-7)
Supplement: Supplementary file 6 — Suppl. Table 5 [file 41416_2019_396_MOESM6_ESM.docx]

**Supplementary Table 5**:

Immunohistochemistry cohort: Multivariate Cox analysis for cytoplasmic expression in tumour cells in patients with ccRCC (Endpoint: overall survival)

|  | **Hazard ratio (HR)** | **95% CI** | **p-level** |
| --- | --- | --- | --- |
| **APLNR cytoplasmic expression at tumour cells** |  |  |  |
| Intensity 0-1-2 | 1.0 | - | - |
| Intensity 3 | 1.7 | 1.0-2.8 | 0.041 |
| **Histological grade (WHO 2016)** |  |  |  |
| G1 | 1.0 | - | - |
| G2 | 1.4 | 0.8-2.4 | 0.247 |
| G3 | 1.6 | 0.9-3.0 | 0.141 |
| G4 | 2.3 | 1.1-4.8 | 0.021 |
| **pT-stage** |  |  |  |
| pT1 | 1.0 | - | - |
| pT2 | 1.3 | 0.6-2.7 | 0.511 |
| PT3-4 | 2.3 | 1.5-3.5 | 0.0001 |
| **R-status** |  |  |  |
| R0 | 1.0 | - | - |
| R1 | 2.5 | 1.2-5.4 | 0.015 |
| **ECOG performance status** |  |  |  |
| ECOG 0 | 1.0 | - | - |
| ECOG 1 | 2.0 | 1.3-2.9 | 0.0009 |
| ECOG 2 | 4.3 | 1.8-10.5 | 0.001 |

Comments: ccRCC, clear-cell renal cell carcinoma (RCC).
