# Supplementary figures and images for: Apelin and apelin receptor expression in renal cell carcinoma
Source: Br J Cancer. 2019 Feb 20;120(6):633–9. doi: 10.1038/s41416-019-0396-7 (PMC6461937; doi:10.1038/s41416-019-0396-7)

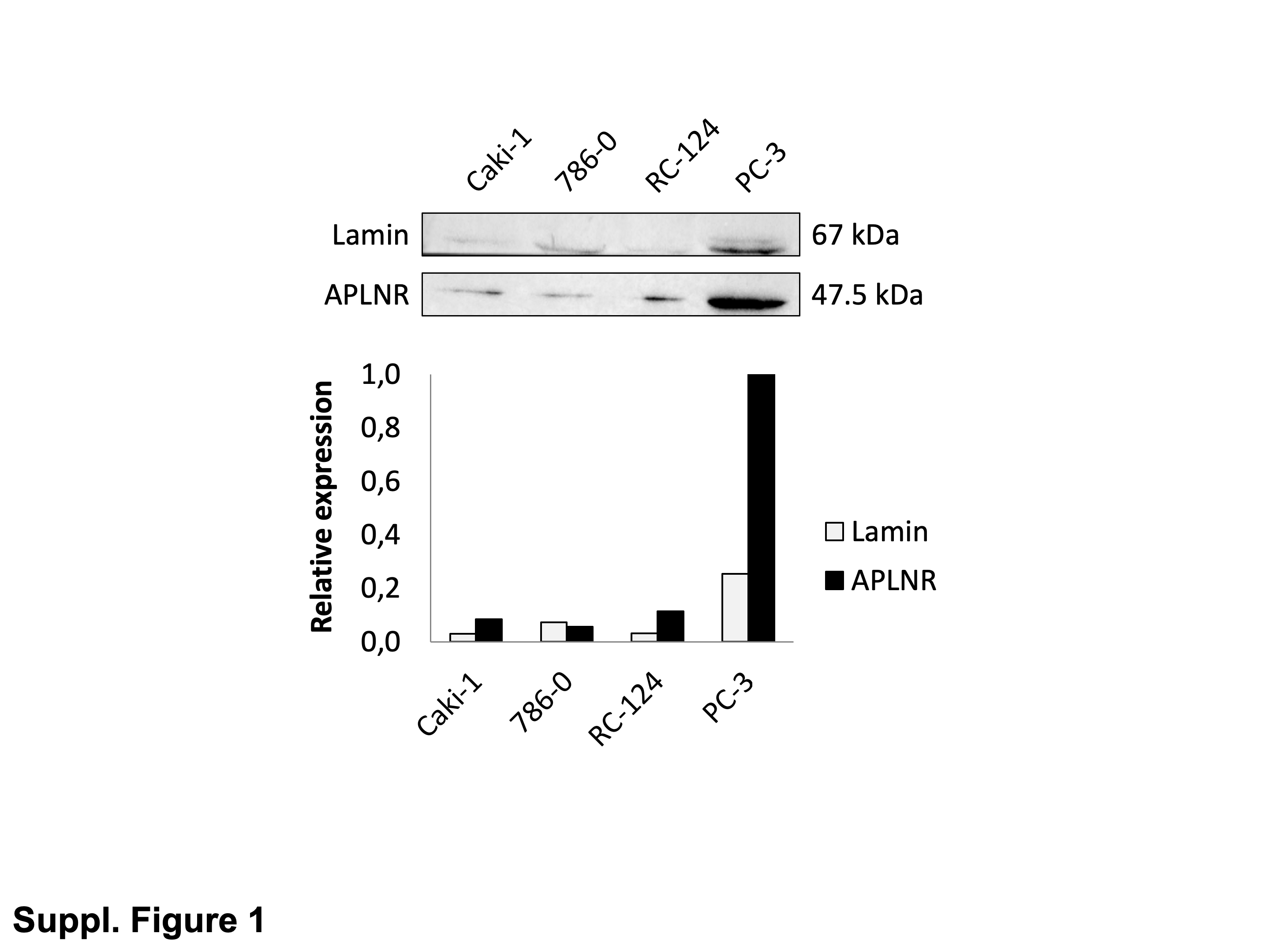

Supplement: Supplementary file 7 — Suppl Figure 1 [file 41416_2019_396_MOESM7_ESM.tif]

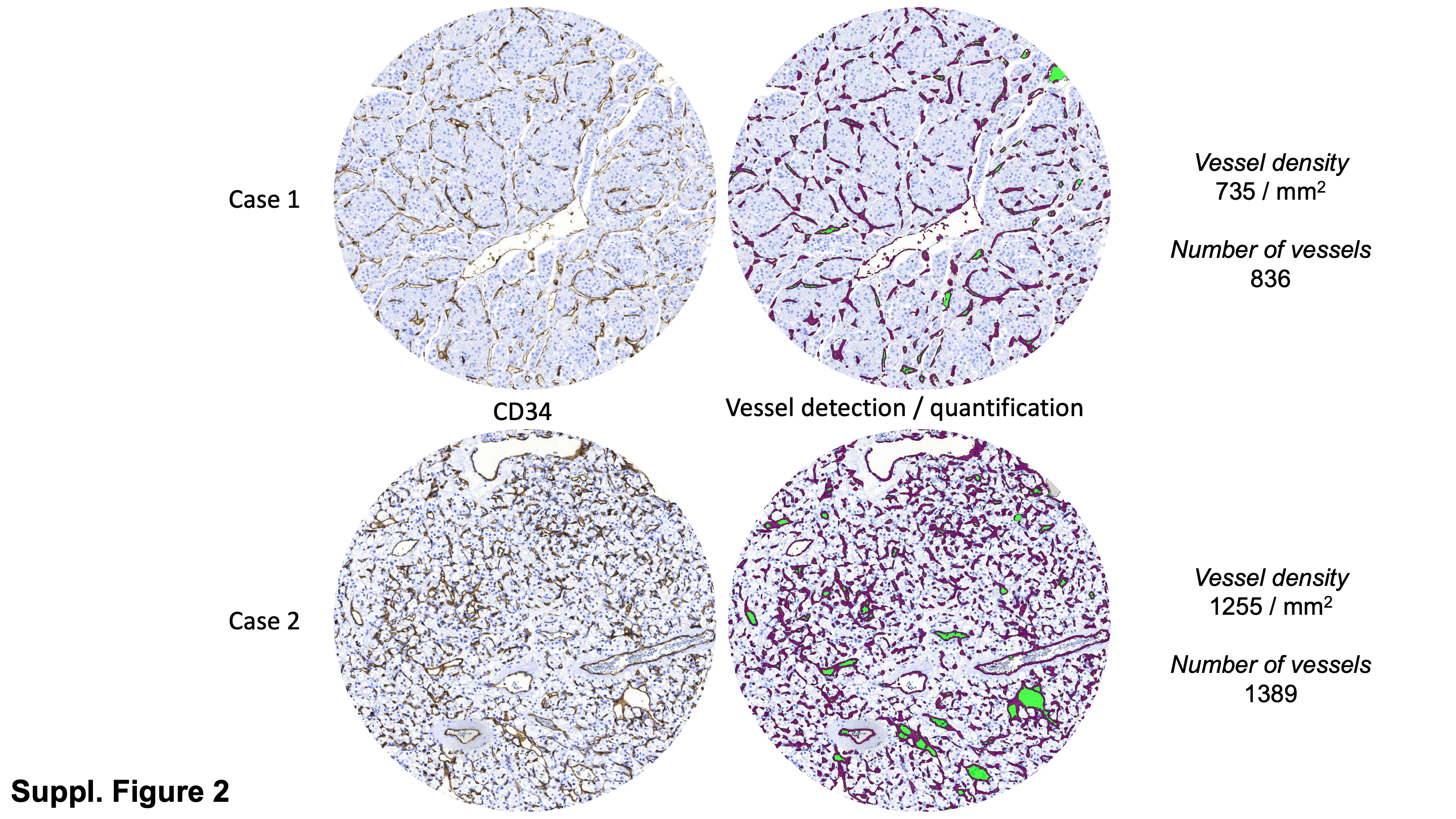

Supplement: Supplementary file 8 — Suppl Figure 2 [file 41416_2019_396_MOESM8_ESM.tif]
